# Supplementary material for: CRISPR-based targeted haplotype-resolved assembly of a megabase region
Source: Nat Commun. 2023 Jan 3;14:22. doi: 10.1038/s41467-022-35389-w (PMC9810730; doi:10.1038/s41467-022-35389-w)
Supplement: Supplementary file 1 — Supplementary Information [file 41467_2022_35389_MOESM1_ESM.pdf]

CRISPR-based targeted haplotype-resolved assembly of a megabase region

Taotao Li[1,2,#], Duo Du[1,2,#], Dandan Zhang[1,2,#], Yicheng Lin[1,2,#], Jiakang Ma[1,2], Mengyu Zhou[1,2], Weida Meng[1,2], Zelin Jin[1,2], Ziqiang Chen[1,2], Haozhe Yuan[1,2], Jue Wang[1,2], Shulong Dong[1,2], Shaoyang Sun[3], Wenjing Ye[4], Bosen Li[3], Houbao Liu[5], Zhao Zhang[3], Yuchen Jiao[6], Zhi Xie[7], Wenqing Qiu[1,8,*], Yun Liu[1,2,*]

1 MOE Key Laboratory of Metabolism and Molecular Medicine, Department of Biochemistry and Molecular Biology, School of Basic Medical Sciences and Shanghai Xuhui Central Hospital, Fudan University, Shanghai, China

2 State Key Laboratory of Medical Neurobiology and MOE Frontiers Center for Brain Science, Institutes of Brain Science, Fudan University, Shanghai, China

3 MOE Key Laboratory of Metabolism and Molecular Medicine, Department of Biochemistry and Molecular Biology, School of Basic Medical Sciences, Fudan University, Shanghai, China

4 Division of Rheumatology and Immunology, Huashan Hospital, Fudan University, Shanghai, China

5 Department of General Surgery, Zhongshan Hospital, Fudan University, Shanghai, China

6 State Key Laboratory of Molecular Oncology, National Cancer Center/National Clinical Research Center for Cancer/Cancer Hospital, Chinese Academy of Medical Sciences and Peking Union Medical College, Beijing, China

7 State Key Laboratory of Ophthalmology, Zhongshan Ophthalmic Center, Sun Yat-sen University, Guangzhou, China

8 Human Phenome Institute, Zhangjiang Fudan International Innovation Center, Fudan University, Shanghai, China

# These authors contributed equally to this work.

* Correspondence to: Wenqin Qiu, PhD, E-mail: qiuwq@fudan.edu.cn; or Yun Liu, PhD, E-mail: yliu39@fudan.edu.cn

Supplementary Figure. 1

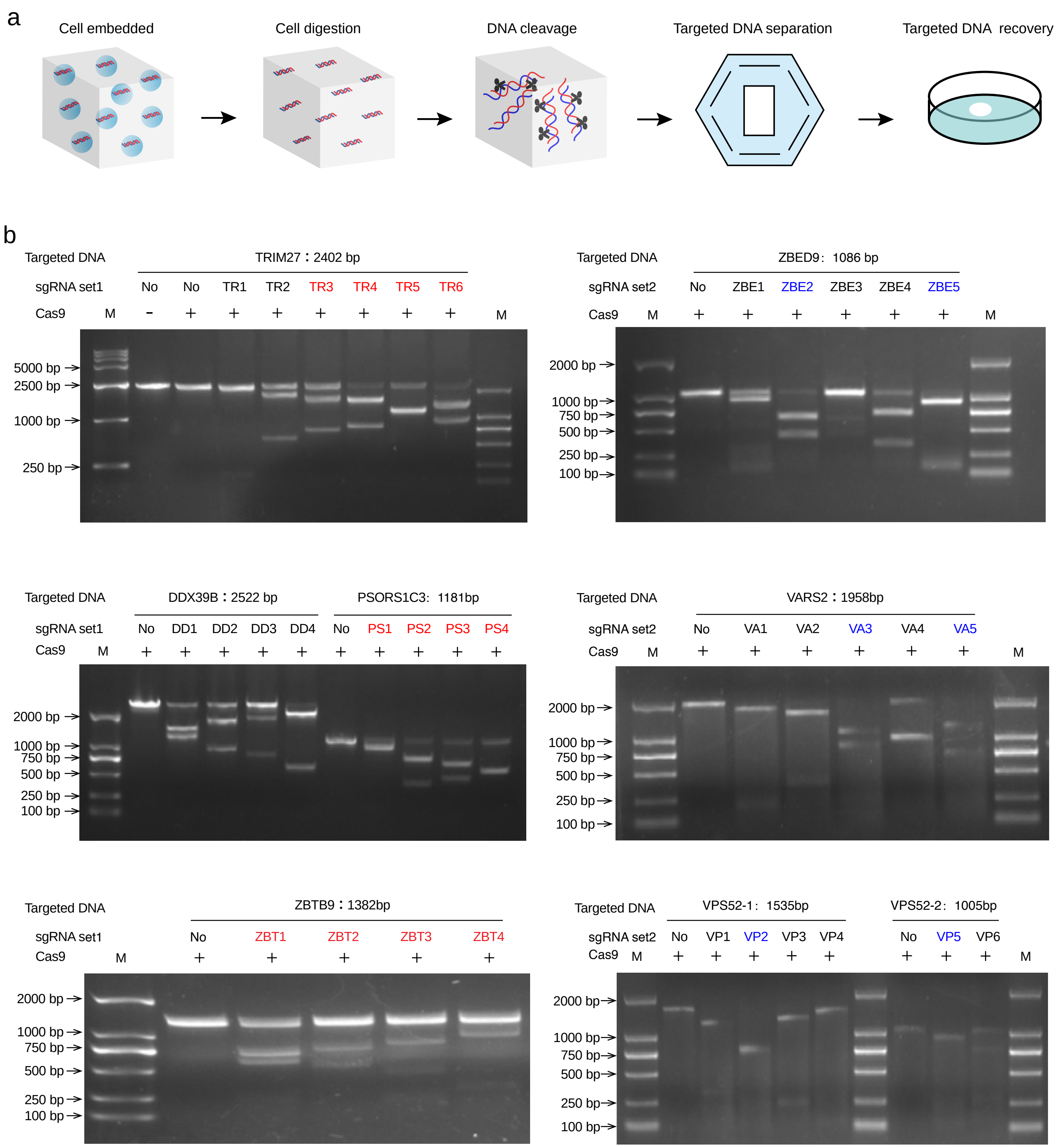

Supplementary Figure. 2

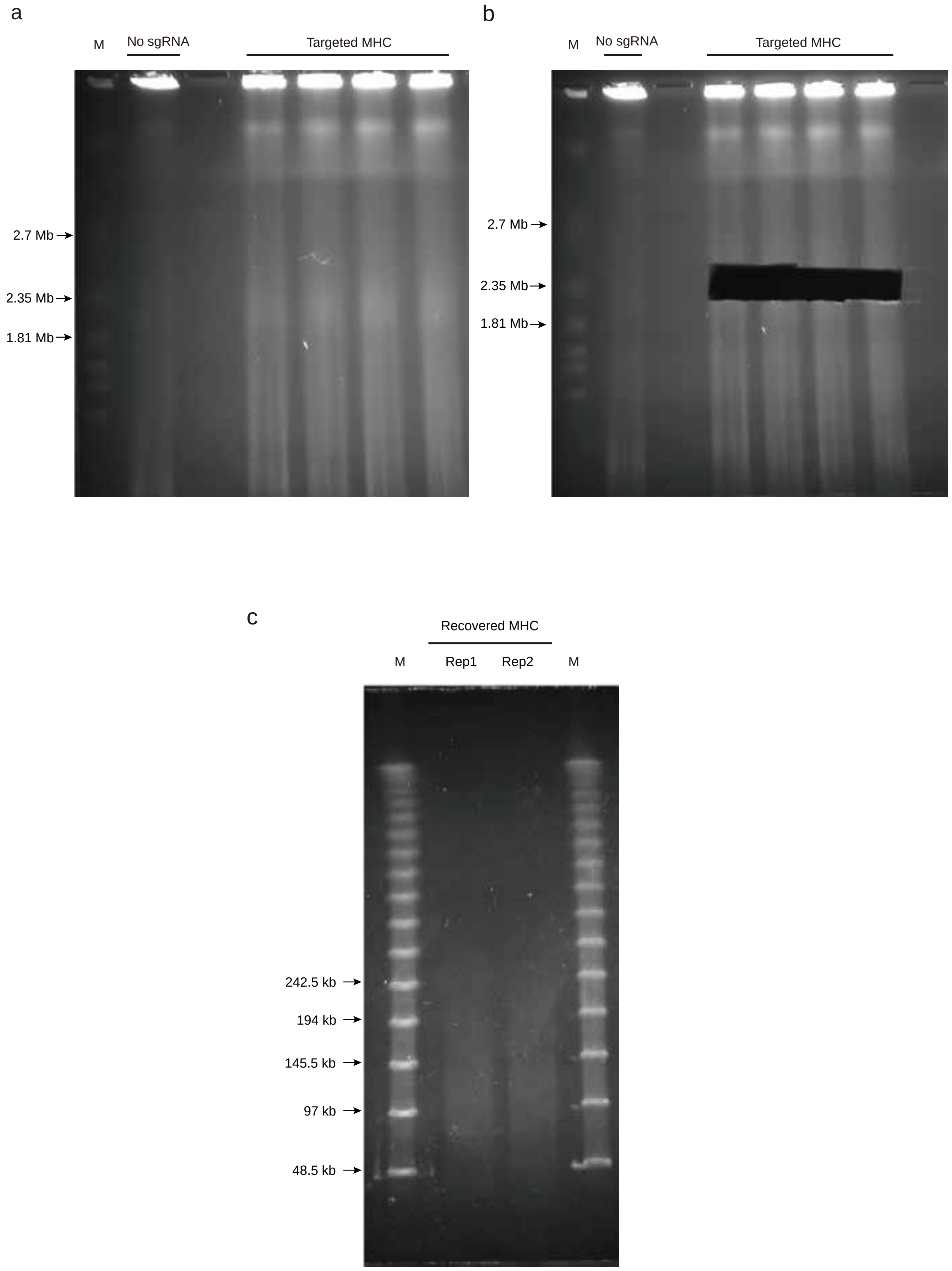

Supplementary Figure. 3

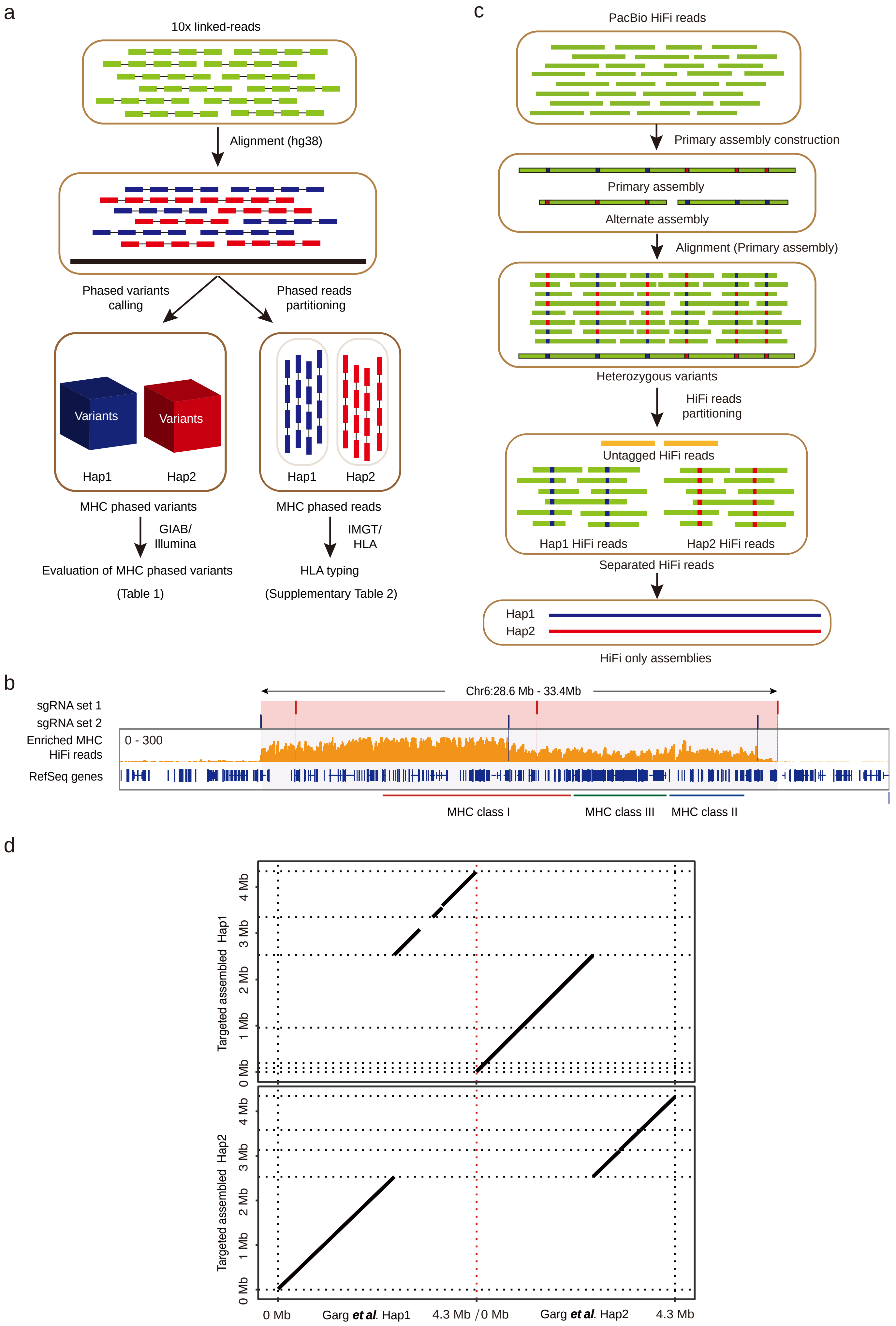

Supplementary Figure. 4

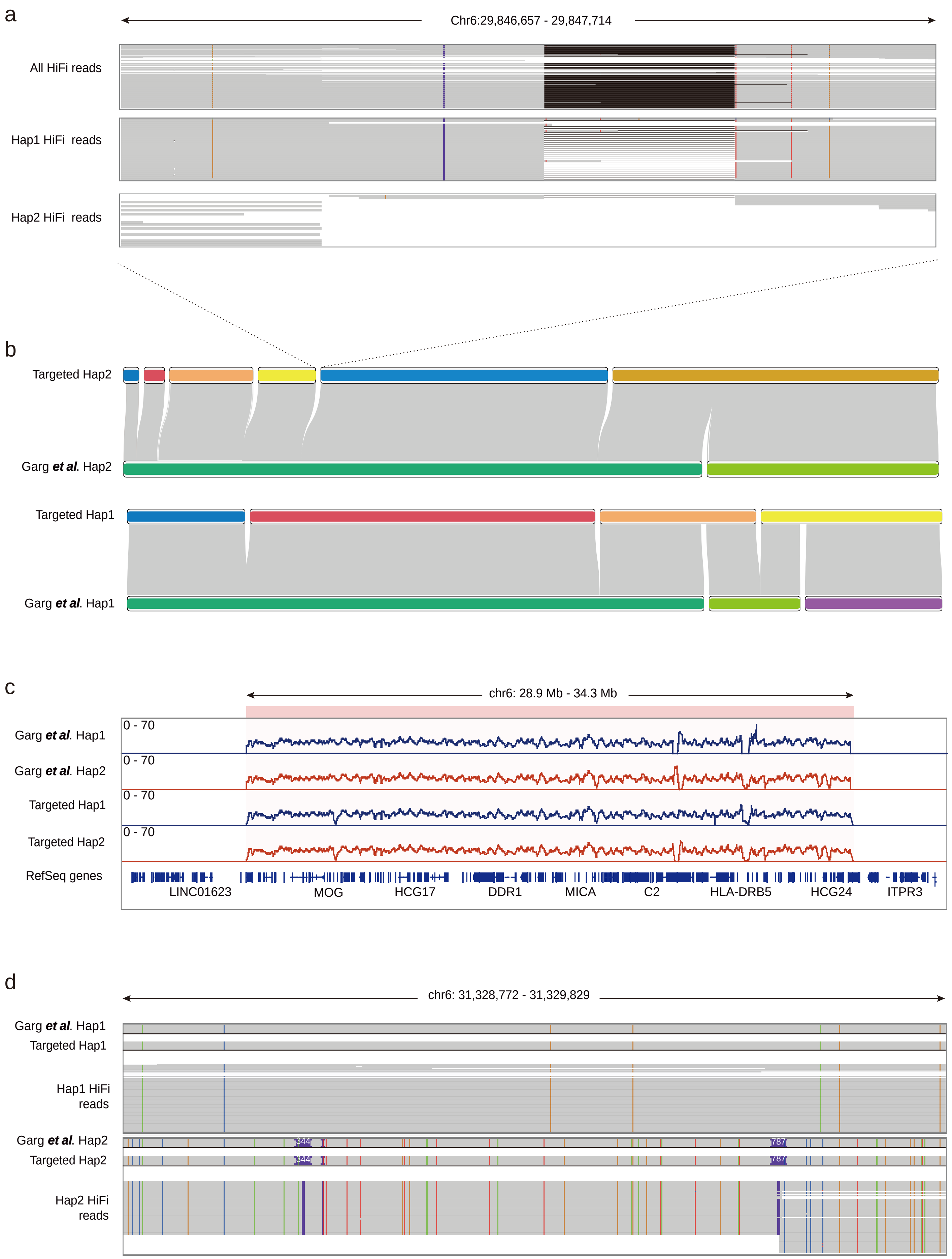

Supplementary Figure. 5

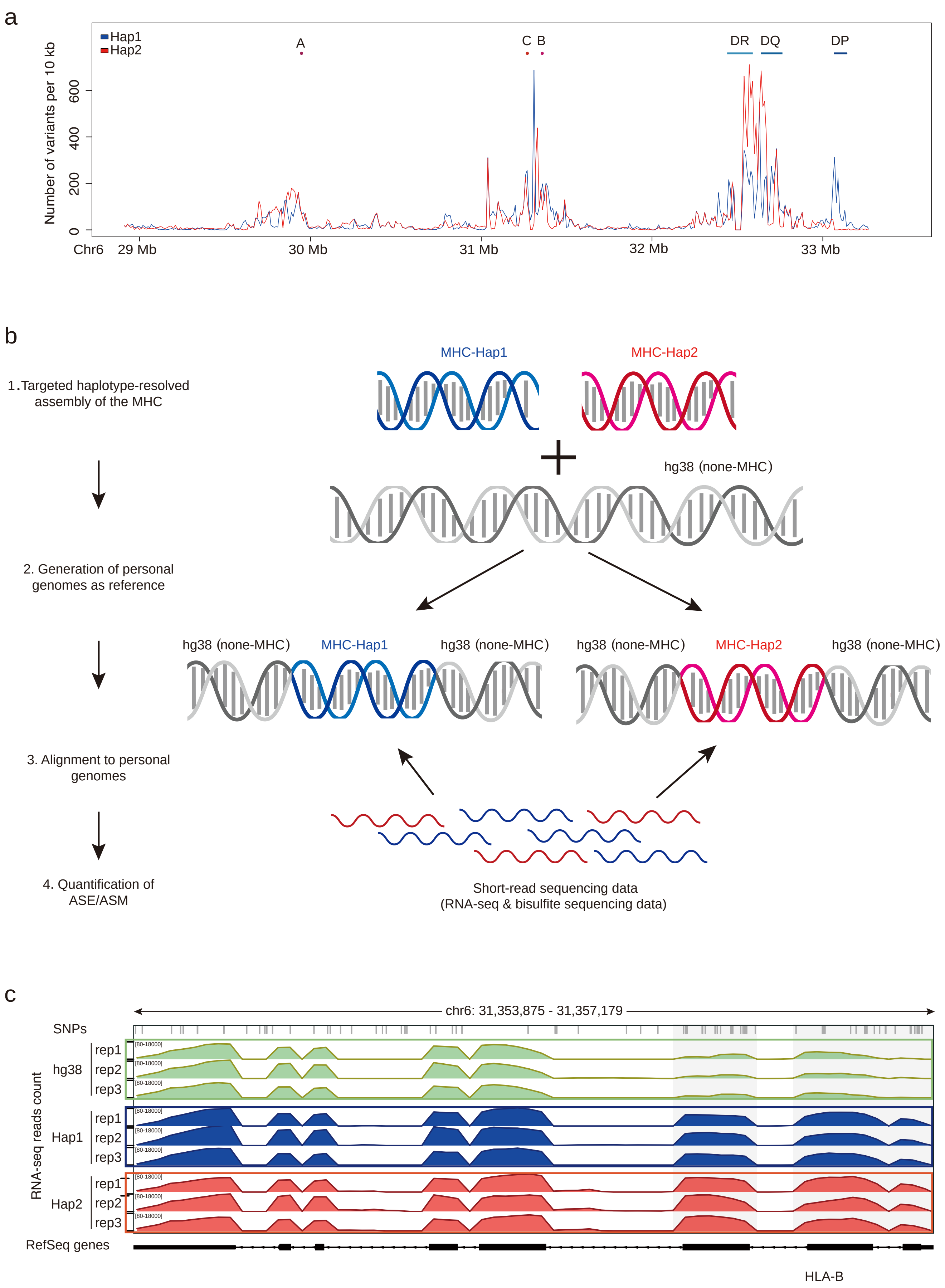

Supplementary Figure. 6

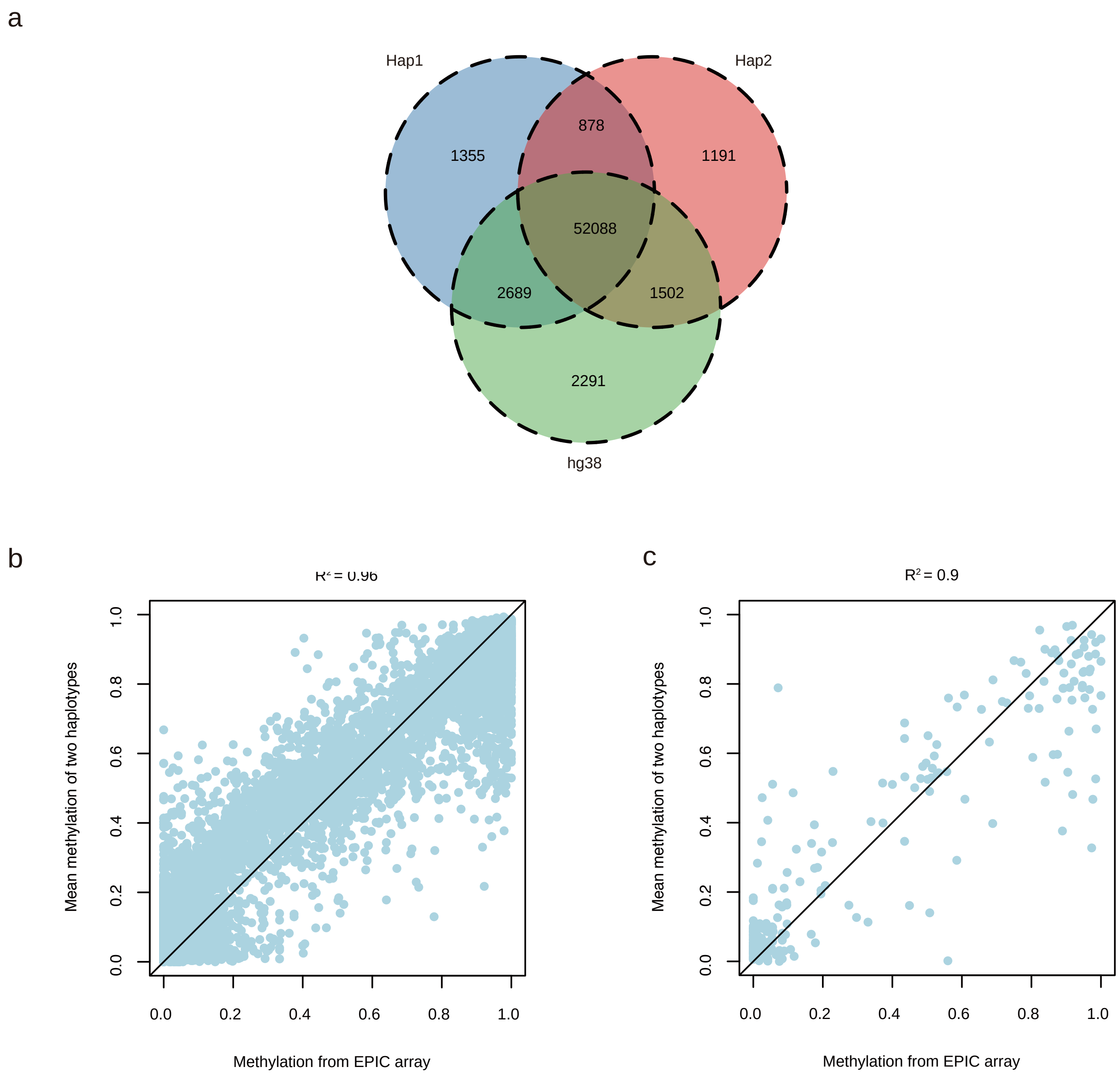

Supplementary Figure. 7

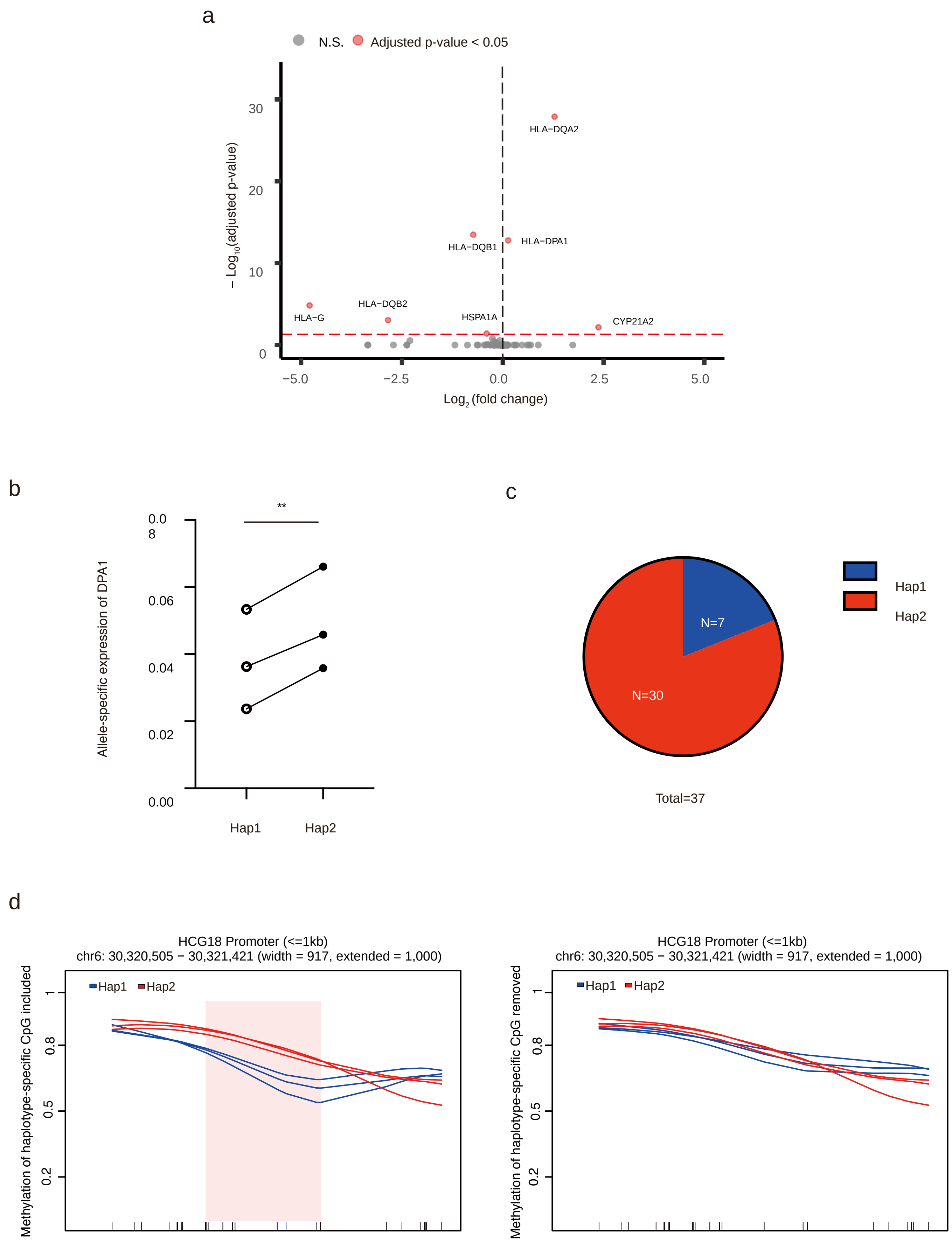

Supplementary Figure. 8

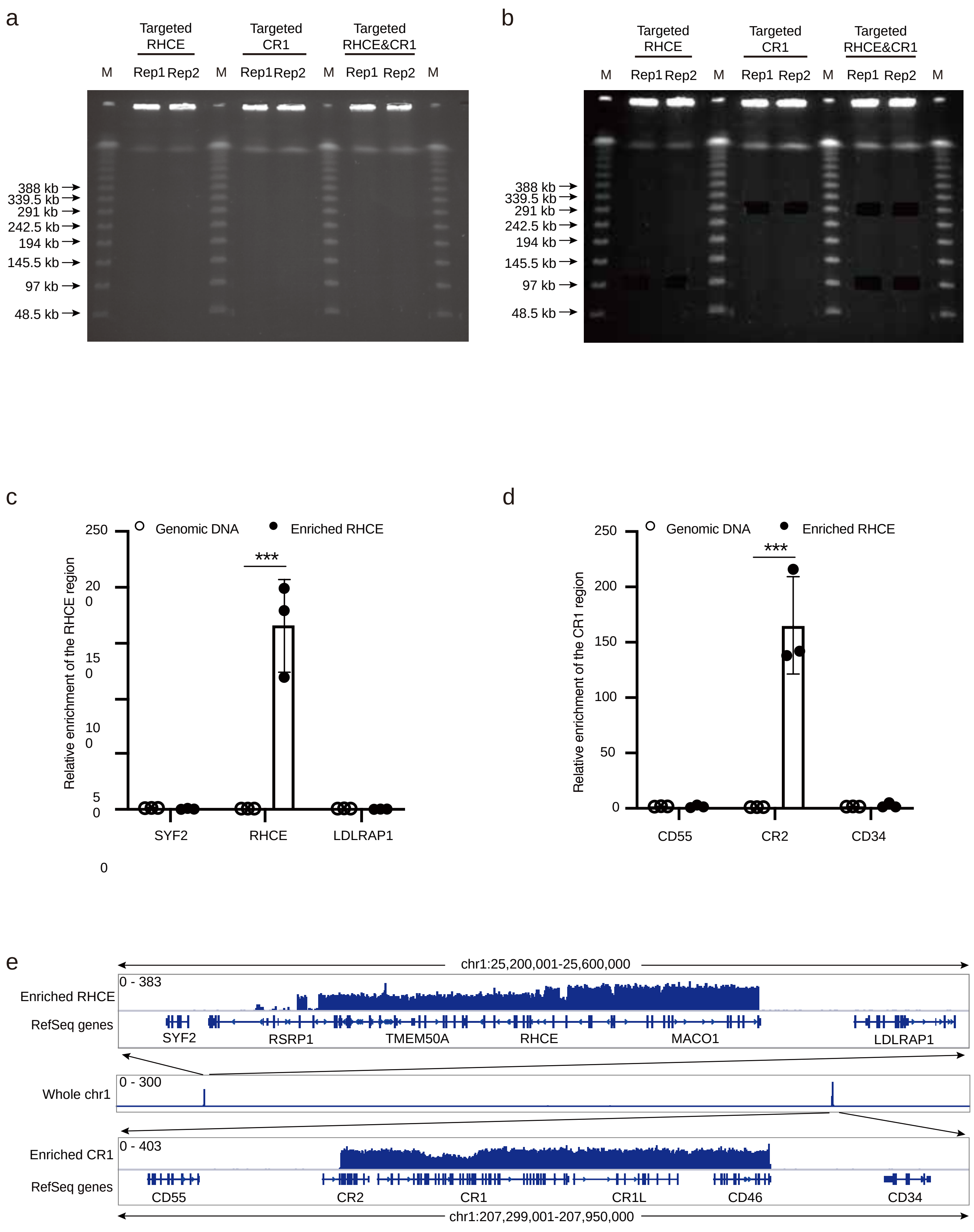

Supplementary Figure. 9

a

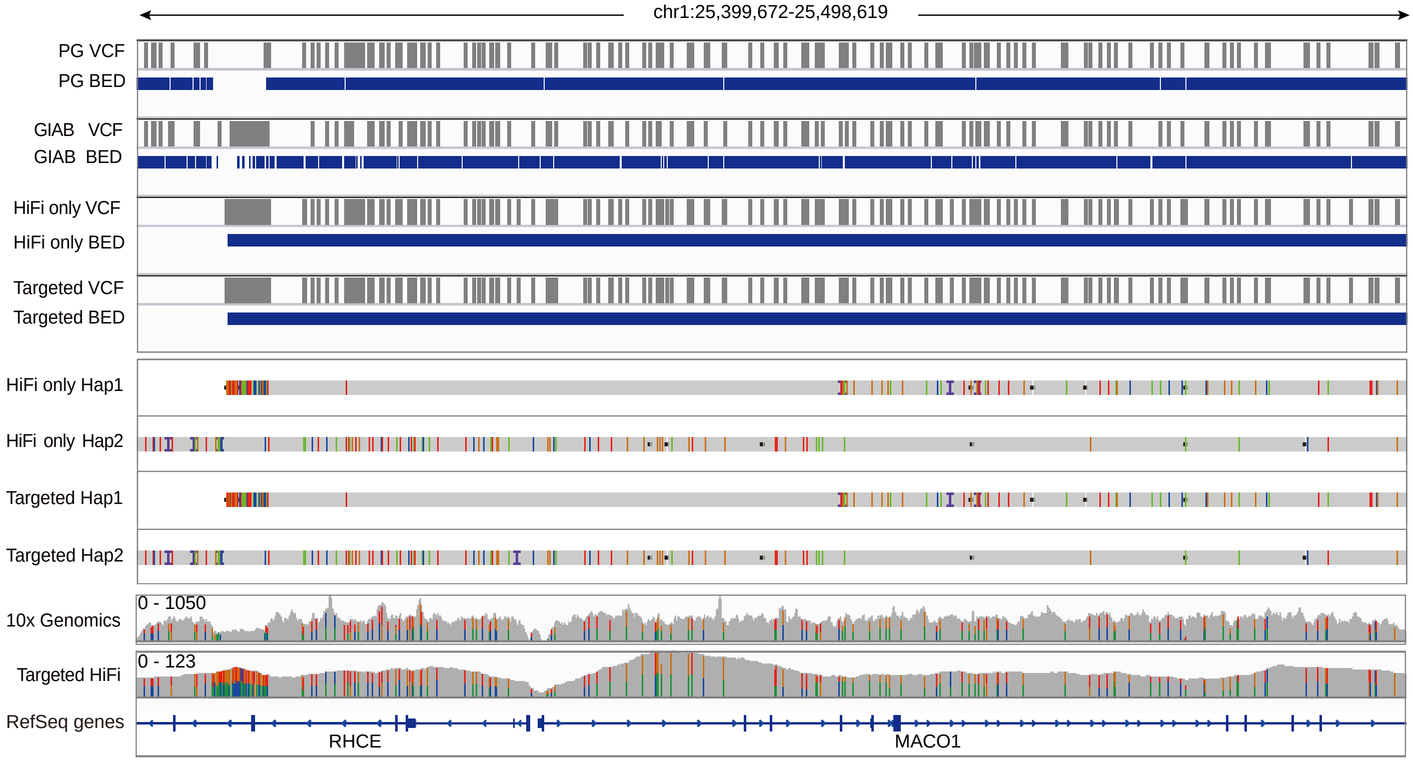

b

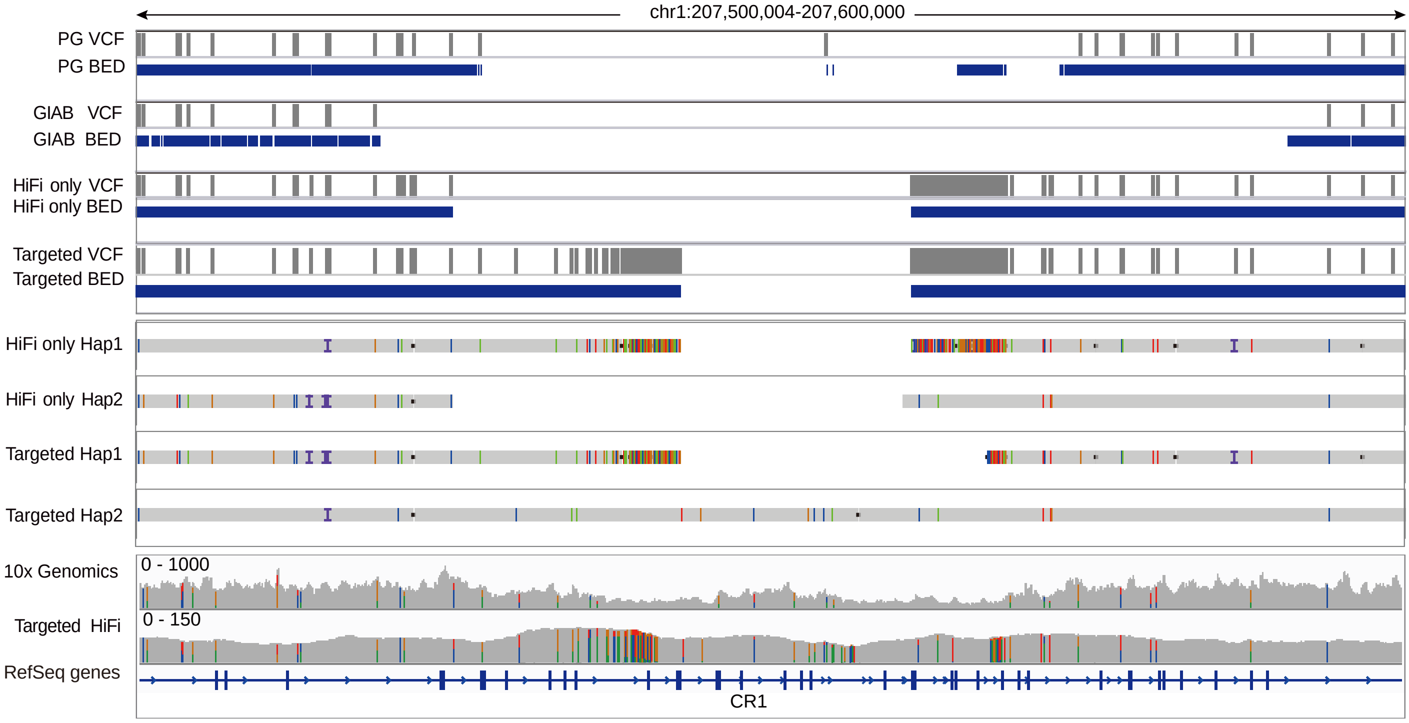

Supplementary Figure. 10

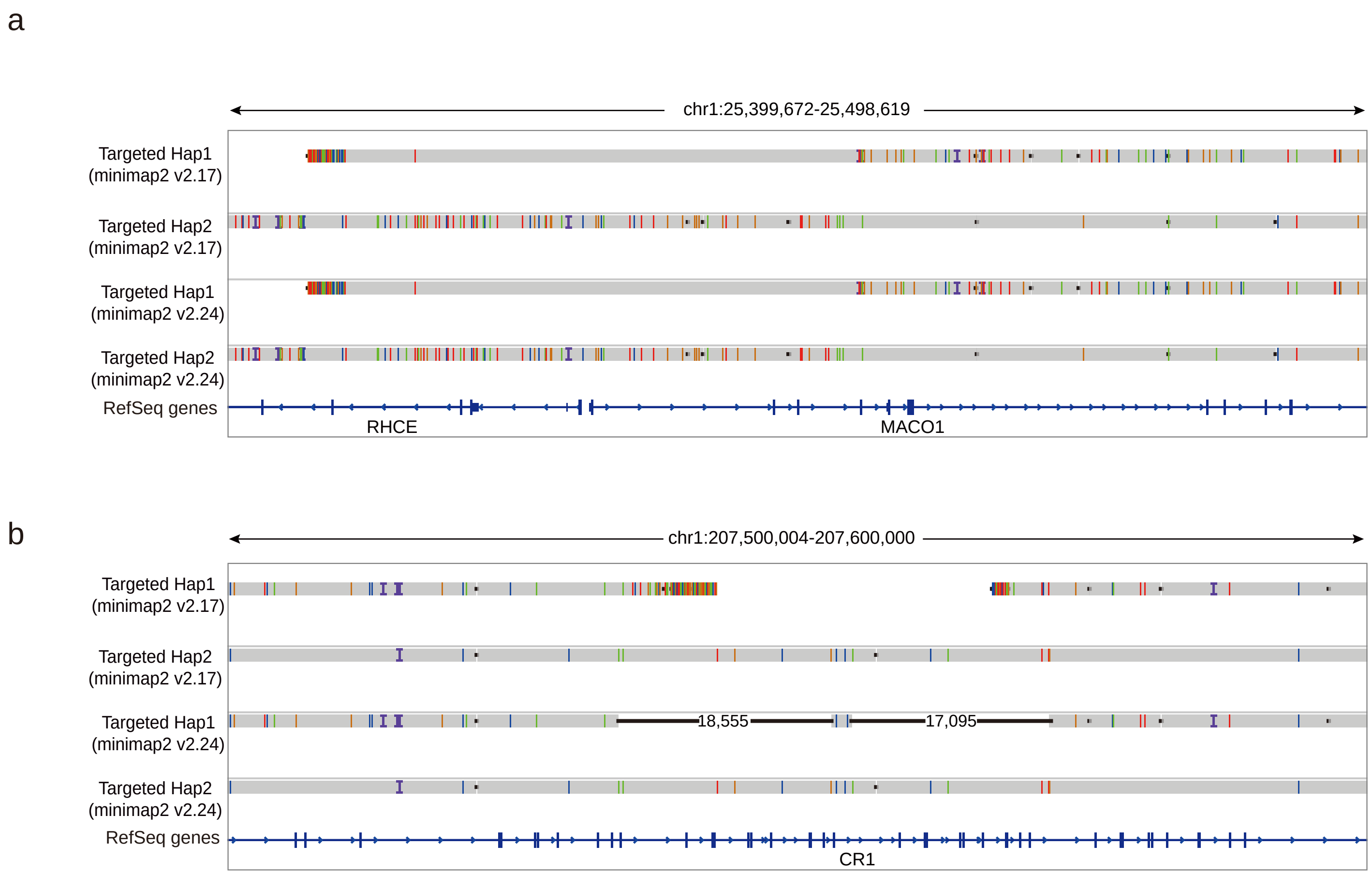

Supplementary Table 1. The performance of variant calling and phasing.

	10x linked-read	HiFi only assembly	Targeted assembly	Garg *et al.*
Homo-variants (GIAB)	6111	6330	6369	6382
Homo-variants (Illumina)	5966	6024	6028	5996
Switch error (GIAB)	0.00%	0.02%	0.00%	0.00%
Switch error (Illumina)	0.42%	0.43%	0.42%	0.43%
Hamming error (GIAB)	0.00%	48.05%	0.00%	0.00%
Hamming error (Illumina)	0.31%	49.67%	0.31%	0.36%

Switch error: the percentage of adjacent SNP pairs wrongly phased in comparison to the benchmarks; Hamming error: the percentage of SNPs wrongly phased in comparison to the benchmarks.

Supplementary Table 2. HLA typing using 10x Genomics linked-reads.

	Alleles typed by phased 10x linked-reads		Alleles predicted by Jain *et al.*	
HLA gene	Hap1	Hap2	Hap1	Hap2
A	11:01:01:01	01:01:01:01	11:01:01G	01:01:01G
B	56:01:01:04	08:01:01:01	56:01:01G	08:01:01G
C	01:148 (...)	07:01:01:01	01:02:01G	07:01:01G
DQA1	01:01:01:01	05:01:01:02	01:01:01G	05:01:01G
DQB1	05:01:01:03	02:01:01:01	05:01:01G	02:01:01G
DRB1	01:01:01:01	03:01:01:01	01:01:01G	03:01:01G

HLA typing for six classical *HLA* genes. For the haplotype 1 of the *HLA-C* gene, several alleles were typed. Information regarding the G' group in HLA alleles predicted by Jain *et al.* is available at 'http://hla.alleles.org/alleles/g_groups.html'.

Supplementary Table 3. Assembly statistics of the targeted MHC region from 10x Genomics linked-reads.

	Hap1	Hap2
**Aligned to the hg38 reference**		
Fraction of the targeted region (%)	93.637	93.8
Total aligned length	4153251	4159618
NGA50	513265	513209
LGA50	3	3
**Without reference**		
Contigs number	18	18

Largest contig length	1930349	1936502
Total length	4267591	4274584

The assembly statistics of the targeted MHC region were calculated by comparing to the hg38 reference with alternate contigs over 10 kb.

Supplementary Table 4. Assembly statistics of the targeted MHC region from PacBio HiFi reads only.

	Hap1	Hap2
**Aligned to the hg38 reference**		
Fraction of the targeted region (%)	97.337	96.533
Total aligned length	4250973	4218050
NGA50	528083	572158
LGA50	3	3
**Without reference**		
Contigs number	6	4
Largest contig length	1572863	2533734
Total length	4341329	4341761

Supplementary Table 5. HLA typing using the assembly result generated from PacBio HiFi reads only.

HLA gene	Hap1	Edit distance	Hap2	Edit distance
A	01:01:01:01	0	11:01:01:01	0
B	08:01:01:01	0	56:01:01:04	0
C	07:01:01:01	0	01:02:01:02	1
DQA1	01:01:01:01	0	05:01:01:02	0
DQB1	05:01:01:03	0	02:01:01:01	0
DRB1	01:01:01:01	0	03:01:01:01	0

Edit distance: The number of different nucleotides between our assemblies and the reported HLA alleles from the IMGT/HLA database. HLA alleles with wrong phasing information are marked in red.

Supplementary Table 6. The breakpoints in the targeted assembled MHC haplotypes relative to the hg38 reference.

		Position in the hg38	Number of aligned HiFi reads
Haplotype1	gap1	chr6: 29529321-29541325	0
	gap2	chr6: 31427070-31428511	2
	gap3	chr6: 32285279-32287741	0
Haplotype2	gap1	chr6: 28971726-28972687	0
	gap2	chr6: 29084414-29084601	0
	gap3	chr6: 29532743-29541325	0
	gap4	chr6: 29846917-29847455	3
	gap5	chr6: 31426738-31430079	5

Supplementary Table 7. Variant accuracy in the targeted assembled MHC region.

	Compared to the BED file from the GIAB						
	Type	Recall	Precision	F1 Score	TP	FN	FP
HiFi only assemblies	SNPs	0.99455	0.995612	0.940356	19526	107	86
	Indels	0.957572	0.923747	0.995081	1625	72	140
Targeted assemblies	SNPs	0.996332	0.996228	0.99628	19556	72	74
	Indels	0.958751	0.928884	0.943581	1627	70	130
Garg *et al.*	SNPs	0.995822	0.991321	0.993567	19546	82	171
	Indels	0.960519	0.916532	0.93801	1630	67	155
	Compared to the BED file from the Illumina PG						
	Type	Recall	Precision	F1 Score	TP	FN	FP
HiFi only assemblies	SNPs	0.985764	0.963162	0.887699	19388	280	742
	Indels	0.973306	0.815933	0.974332	1896	52	439
Targeted assemblies	SNPs	0.987492	0.963749	0.975476	19422	246	731
	Indels	0.974846	0.820286	0.890912	1899	49	427
Garg *et al.*	SNPs	0.983018	0.960433	0.971594	19334	334	797
	Indels	0.973819	0.813545	0.886496	1897	51	446

Recall = TP/(TP+FN)

Precision = TP/(TP+FP)

F1 Score = 2 * Precision * Recall / (Precision + Recall)

true-positives (TP): variants/genotypes that match in truth and query.

false-positives (FP): variants that have mismatching genotypes or alt alleles, as well as query variant calls in regions a truth set would call confident hom-ref regions.

false-negatives (FN): variants present in the truth set, but missed in the query.

Supplementary Table 8. Assembly statistics of the targeted *RHCE* and *CR1* regions.

		hap1		hap2	
		Contig number	Sum-length(bp)	Contig number	Sum-length(bp)
HiFi only assemblies	*RHCE*	1	92502	1	99067
	CR1	1	290788	2	291626
Targeted assemblies with both 10x and HiFi	*RHCE*	1	92502	1	99070
	CR1	1	291383	1	326354

Supplementary Table 9. The range of DNA yields from one gel plug.

Targeted region	Low recovery rate			High recovery rate		
	Concentration (ng/ul)	Volume (ul)	Amounts (ng)	Concentration (ng/ul)	Volume (ul)	Amounts (ng)

MHC	~0.08	~50	~4	~0.35	~30	~10
RHCE	~0.03	~20	~0.6	~0.08	~40	~3.2
CR1	~0.04	~50	~2	~0.12	~25	~3

Supplementary Table 10. Running time/peak memory and cost per sample with the use of our targeted approach, compared to Garg *et al.* and the two other haplotype-resolved assembly methods.

	Targeted assemblies	Whole genome assembles (Garg *et al.*)	Trio-binning (S Koren *et al.*)	MHC benchmark (Chin CS *et al.*)
Reagent cost	~ 275	~ 415	~ 147	~ 497
Sequencing cost	~ 2594	~ 12801	~ 27971	~ 12842
Total cost (USD)	~ 2869	~ 13216	~ 28118	~ 13339
CPU time(h)	~ 415	~ 1440	~ 57292	~ 1170
Elapsed time(h)	~ 26	~ 106	~ 1005	~ 77
Peak memory (Gb)	~ 29	~ 90	~ 511	~ 103

All tools were benchmarked on a computational node with 72 CPUs, with CentOS Linux release 7.4.1708 (Core) and 512 GB of random-access memory.

Supplementary Figure 1. The CRISPR-based targeted enrichment of a megabase region

a. Schematics of the CRISPR-based targeted enrichment. Cells were embedded in agarose plugs and digested with proteinase K. The targeted genomic region was cleaved by CRISPR-based in-gel digestion and separated by PFGE. The final targeted HMW MHC molecules were recovered through dialysis. **b**. The cleavage efficiencies of the designed sgRNAs were evaluated individually. The efficiency was evaluated through *in vitro* cleavage assays with PCR products amplified from the targeted regions. Two sets of sgRNAs chosen for the final targeted enrichment of the MHC region are highlighted with red and blue texts, respectively. The experiments were repeated at least three times, and one representative image is shown.

Supplementary Figure 2. Targeted separation and recovery of the HMW MHC molecules

a. The targeted MHC region cleaved by CRISPR-based in-gel digestion was separated by PFGE. The experiments were repeated at least three times, and one representative image is shown. **b.** The bands corresponding to ~ 2.3Mb indicated by the *H. wingei* CHEF DNA Size Markers (M) were cut out of the gel for the enrichment of HMW MHC molecules. The experiments were repeated at least three times, and one representative image is shown. **c.** The final enriched HMW DNA molecules recovered from LMP agarose gels were still longer than 50 kb as indicated by the CHEF DNA size standards-Lambda Ladder (M). The experiments were repeated at least three times, and one representative image is shown.

Supplementary Figure 3. Analyses with 10x Genomics linked-read data or PacBio HiFi reads

a. Schematics of phased variant calling and HLA typing with 10x Genomics linked-read data. **b.** The coverage of the PacBio HiFi reads on the targeted MHC region is shown. The digestion positions for two sets of sgRNAs are indicated as red and blue bars, respectively. **c.** Schematics of targeted haplotype-resolved assembly of the MHC region with PacBio HiFi reads only. **d.** The comparison between our assembly result with PacBio HiFi reads only and the MHC region from genome assembly reported previously (Garg et al.) for each haplotype. The Y-axis indicates the coordinate of our targeted assembly, and the X-axis indicates the coordinate of Garg *et al.*

Supplementary Figure 4. Targeted haplotype-resolved assemblies of the MHC region with both 10x Genomics linked-read data and PacBio HiFi reads

a. An example of the coverage of the PacBio HiFi reads on one breakpoint in our targeted assemblies for each haplotype. The purple dots indicate small insertions (<50 bp) compared to the hg38 reference. Black lines indicate deletions greater than 50 bp compared to the hg38 reference. **b**. The comparison of our targeted assemblies and genome assemblies reported previously (Garg *et al.*) shows high consistency across the targeted MHC region for each haplotype. The consistent assemblies are indicated as grey lines. The different colored horizontal bars represent contigs. **c.** Collapsed analyses for the targeted MHC region in each assembly. Blue and red lines represent the coverage of downloaded PacBio HiFi reads of GM12878 cells from the GIAB

aligned to the haplotype 1 or haplotype 2 of different assemblies. The pink bar on the top indicates the targeted MHC region. **d.** A region with two insertions (344 bp and 787 bp) was identified and supported by the presence of PacBio HiFi reads.

Supplementary Figure 5. Functional genomics analyses with the targeted haplotype-resolved personal assemblies

a. The density plot of genetic variants throughout the targeted MHC region. The X-axis indicates the coordinates of the targeted MHC assemblies and the Y-axis indicates the number of genetic variants (SNPs and InDels) relative to hg38 reference in each 10 kb window. Blue line: the assembly of haplotype 1; red line: the assembly of haplotype 2. Red dots: locations of three classical HLA I genes; blue bars: locations of three classical HLA II genes. **b.** Schematics of analyses of short-read sequencing data using personal genome references. **c**. Quantification of RNA-Seq reads on the *HLA-B* gene based on difference references (indicated at the left). Three replicates are shown. Two regions with high density of SNPs which have biased sequence alignment are indicated as grey bars.

Supplementary Figure 6. DNA methylation analyses with the targeted haplotype-resolved personal assemblies

a. The Venn diagram shows the numbers of CpGs specific or shared by the assembly of each haplotype and the hg38 reference. **b** and **c**. The methylation correlations between the EPIC array and bisulfite sequencing data. We analyzed the methylation level for shared CpGs between the Illumina Infinium EPIC methylation array and bisulfite sequencing data. The shared CpGs were separated into two groups by

whether genetic variants are present within 5 bp of the interrogated CpG site on the probes of EPIC array (**b**), or absent from the probes (**c**).

Supplementary Figure 7. Analyses of allele-specific expression and methylation

a. The volcano plot of genes that are differentially expressed between two alleles. The red line represents the cut-off for statistical significance of adjusted P-value less than 0.05 (Wald test, two-sided). Red dots indicate the allele-specific expressed genes. **b.** Allele specific expression of *HLA-DPA1* gene was quantified by qRT-PCR with haplotype-specific primers for the GM12878 cells. Data are represented from three independent experiments, $P = 0.0074$ (** $0.001 < P < 0.01$, paired student's t-test, two-sided). Source data are provided as a Source Data file. **c.** The number of clones belonging to two haplotypes from amplified PCR product of cDNA of *HLA-DPA1* gene for the GM12878 cells using Sanger sequencing. **d**. The allele-specific methylation analyses with (the left panel) or without (the right panel) the haplotype-specific CpGs included. The methylation level of the promoter region of *HCG18* gene is shown as an example. The identified DMR indicating allele-specific methylation is indicated with the pink box. The shared CpGs between two haplotypes are shown as black short lines, while haplotype-specific CpGs are indicated as blue or red short lines.

Supplementary Figure 8. Targeted enrichment of two challenging medically relevant genes (*RHCE* and *CR1*)

a. The targeted *RHCE* or *CR1* region was separated by PFGE after cleaved by CRISPR-based in-gel digestion separately or together. The experiments were repeated

at least three times, and one representative image is shown. **b**. The bands corresponding to ~ 100 kb and ~ 300 kb indicated by the CHEF DNA size standards Lambda Ladder (M) were cut out of the gel for the enrichment of HMW *RHCE* and *CR1* molecules respectively. The experiments were repeated at least three times, and one representative image is shown. **c-d.** QPCR analyses showed significant enrichment of the targeted *RHCE* (c) or *CR1* (d) region. Data are represented as mean ± SEM from three independent experiments, *P* values: *RHCE P* = 0.00237, *CR2 P* = 0.00293 (****P* < 0.001, student's t-test, two-sided). Source data are provided as a Source Data file. **e.** Illumina short-read sequencing data of enriched HMW molecules, in which the targeted *RHCE* and *CR1* regions were isolated together, shows the successful enrichment of both *RHCE* and *CR1* regions.

Supplementary Figure 9. Targeted haplotype-resolved assemblies of two challenging medically relevant genes (*RHCE* and *CR1*)

Integrated Genomics Viewer (IGV) showing that newly identified variants from haplotype-resolved assemblies are supported by the presence of PacBio HiFi reads in the targeted *RHCE* (a) or *CR1* (b) region. The colorful lines and dots positioned in the targeted assemblies and HiFi reads indicate four types of nucleotides different from the hg38 reference. The purple character "I" indicate small insertions (<50 bp) compared to the hg38 reference. Black dots indicate small deletions (<50 bp) compared to the hg38 reference. PG: the Illumina Platinum Genomes.

Supplementary Figure 10. The comparison of alignment results with different version of minimap2

The alignments of the targeted assemblies to the hg38 reference were shown with the IGV in the targeted *RHCE* (a) or *CR1* (b) region with two different versions of minimap2 (v2.17 and v2.24).
