# Supplementary material for: CRISPR-based targeted haplotype-resolved assembly of a megabase region
Source: Nat Commun. 2023 Jan 3;14:22. doi: 10.1038/s41467-022-35389-w (PMC9810730; doi:10.1038/s41467-022-35389-w)
Supplement: Supplementary file 3 — Description of Additional Supplementary Files [file 41467_2022_35389_MOESM3_ESM.pdf]

Description of Additional Supplementary Files

Title: Supplementary Data 1
Description: Collapsed region in the assemblies.
a: None corresponding position was found in the hg38 reference.

Title: Supplementary Data 2
Description: Variant accuracy in the region containing *C4A/B, TNXA/B* and *CYP21A2* genes.
Recall = TP/(TP+FN)
Precision = TP/(TP+FP)
F1 Score = 2 * Precision * Recall / (Precision + Recall)
true-positives (TP): variants/genotypes that match in truth and query.
false-positives (FP): variants that have mismatching genotypes or alt alleles, as well as query variant calls in regions a truth set would call confident hom-ref regions.
false-negatives (FN): variants present in the truth set, but missed in the query.

Title: Supplementary Data 3
Description: The genes with allele-specific expression.
A two-sided Wald test p-value was calculated by DESeq2 for two alleles of each gene.

Title: Supplementary Data 4
Description: The allele-specific DNA methylation regions within the MHC region.

Title: Supplementary Data 5
Description: Variant accuracy in the targeted *RHCE* and *CR1* regions compared to the variants in the benchmarks using vcf.
Switch error: the percentage of adjacent SNP pairs wrongly phased in comparison to the benchmarks. Hamming error: the percentage of SNPs wrongly phased in comparison to the benchmarks. FNR: False negative rate, the percentage of true variants that are missed in the assembly. FDR: False discovery rate, the percentage of assembly-based variant calls that are not present in the benchmark.

Title: Supplementary Data 6
Description: Variant accuracy in the targeted *RHCE* and *CR1* regions compared to the variants in the benchmarks using bed.
Recall = TP/(TP+FN)
Precision = TP/(TP+FP)
F1 Score = 2 * Precision * Recall / (Precision + Recall)
true-positives (TP): variants/genotypes that match in truth and query.
false-positives (FP): variants that have mismatching genotypes or alt alleles, as well as query variant calls in regions a truth set would call confident hom-ref regions.
false-negatives (FN): variants present in the truth set, but missed in the query.

Title: Supplementary Data 7

Description: DNA sequences of sgRNAs and PCR primers used in this work.

/5Biosg/ = 5’ biotin added
