# Supplementary material for: CRISPR-based targeted haplotype-resolved assembly of a megabase region
Source: Nat Commun. 2023 Jan 3;14:22. doi: 10.1038/s41467-022-35389-w (PMC9810730; doi:10.1038/s41467-022-35389-w)
Supplement: Supplementary file 11 — Reporting Summary [file 41467_2022_35389_MOESM11_ESM.pdf]

Corresponding author(s): Yun Liu
Last updated by author(s): Nov 17, 2022

Reporting Summary

Nature Portfolio wishes to improve the reproducibility of the work that we publish. This form provides structure for consistency and transparency in reporting. For further information on Nature Portfolio policies, see our Editorial Policies and the Editorial Policy Checklist.

Statistics

For all statistical analyses, confirm that the following items are present in the figure legend, table legend, main text, or Methods section.

n/a	Confirmed	
☐	☒	The exact sample size (*n*) for each experimental group/condition, given as a discrete number and unit of measurement
☐	☒	A statement on whether measurements were taken from distinct samples or whether the same sample was measured repeatedly
☐	☒	The statistical test(s) used AND whether they are one- or two-sided<br>*Only common tests should be described solely by name; describe more complex techniques in the Methods section.*
☐	☒	A description of all covariates tested
☐	☒	A description of any assumptions or corrections, such as tests of normality and adjustment for multiple comparisons
☐	☒	A full description of the statistical parameters including central tendency (e.g. means) or other basic estimates (e.g. regression coefficient) AND variation (e.g. standard deviation) or associated estimates of uncertainty (e.g. confidence intervals)
☐	☒	For null hypothesis testing, the test statistic (e.g. *F*, *t*, *r*) with confidence intervals, effect sizes, degrees of freedom and *P* value noted<br>*Give P values as exact values whenever suitable.*
☒	☐	For Bayesian analysis, information on the choice of priors and Markov chain Monte Carlo settings
☒	☐	For hierarchical and complex designs, identification of the appropriate level for tests and full reporting of outcomes
☐	☒	Estimates of effect sizes (e.g. Cohen's *d*, Pearson's *r*), indicating how they were calculated

Our web collection on statistics for biologists contains articles on many of the points above.

Software and code

Policy information about availability of computer code

Data collection	No software is used for data collection.
Data analysis	Open source software and code: proc10xG (process_10xReads.py v0.0.2, regen_10xReads.py v0.0.1, filter_10xReads.py v0.0.1)(https://github.com/CeciliaDeng/proc10xG), Bwa mem v0.7.15-r1140, Seqkit v0.10.0, Long Ranger v.2.2.2, WhatsHap v0.17, Supernova assembler v2.1.1, Pbmarkdup v1.0.0, Minimap2 v2.17-r941, Minimap2 v2.17, Minimap2 v2.24, DeepVariants v0.10.0, Hifiasm v0.11-r302, MUMmer v4.0.0beta2, bowtie2 v2.2.3, bismark v0.22.1, bsseq v1.24.4, minfi package v1.28.0, liftover(https://genome.ucsc.edu/cgi-bin/hgLiftOver), fastp v0.21.0, Trimmomatic v0.39, HISAT2 2.1.0, WASP package(https://github.com/bmvdgeijn/WASP), DESeq2 v1.38.0, samtools v1.9, Hap.py v0.3.15, Racon v1.4.20, Dipcall-0.1,longranger v2.2.2, gatk3.8, HLA-VBseq v2.

For manuscripts utilizing custom algorithms or software that are central to the research but not yet described in published literature, software must be made available to editors and reviewers. We strongly encourage code deposition in a community repository (e.g. GitHub). See the Nature Portfolio guidelines for submitting code & software for further information.

Data

Policy information about availability of data

All manuscripts must include a data availability statement. This statement should provide the following information, where applicable:

- Accession codes, unique identifiers, or web links for publicly available datasets
- A description of any restrictions on data availability
- For clinical datasets or third party data, please ensure that the statement adheres to our policy

All relevant data were generated from GM12878 cells. All relevant data supporting the key findings of this study are available within the article and its Supplementary Information files or from the corresponding authors upon reasonable request. The targeted assembled haplotypes and called variants can be downloaded from: [https://liulab.fudan.edu.cn/targeted_assemblies.html]. DNA sequencing data with the Illumina short-read platform (accession code no. SRR17250933 [https://www.ncbi.nlm.nih.gov/sra/?term=SRR17250933] for whole-genome sequencing; accession code no. SRR17250934 [https://www.ncbi.nlm.nih.gov/sra/?term=SRR17250934] for the targeted enriched MHC region; accession code no. SRR21079439 [https://www.ncbi.nlm.nih.gov/sra/?term=SRR21079439] for the targeted enriched RHCE and CR1 regions), 10x Genomics linked-read (accession code no. SRR17250932 [https://www.ncbi.nlm.nih.gov/sra/?term=SRR17250932] for the targeted enriched MHC region; accession code no. SRR21079437 [https://www.ncbi.nlm.nih.gov/sra/?term=SRR21079437] for the targeted enriched RHCE region; accession code no. SRR21079438 [https://www.ncbi.nlm.nih.gov/sra/?term=SRR21079438] for the targeted enriched CR1 region), the PacBio HiFi long-read (accession code no. SRR17250931 [https://www.ncbi.nlm.nih.gov/sra/?term=SRR17250931] for the targeted enriched MHC region; accession code no. SRR21079435 [https://www.ncbi.nlm.nih.gov/sra/?term=SRR21079435] for the targeted enriched RHCE region; accession code no. SRR21079436 [https://www.ncbi.nlm.nih.gov/sra/?term=SRR21079436] for the targeted enriched CR1 region), and RNA-Seq data (accession code no. SRR17250928 - SRR17250930 [https://www.ncbi.nlm.nih.gov/sra/?term=SRR17250928], [https://www.ncbi.nlm.nih.gov/sra/?term=SRR17250929], [https://www.ncbi.nlm.nih.gov/sra/?term=SRR17250930]) were deposited with Sequence Read Archive (SRA). Bisulfite sequencing data from the targeted enriched MHC region and the Illumina methylation EPIC beadchip data from genomic DNA of GM12878 cells are available in the Gene Expression Omnibus (GEO) database under accession number GSE192499 [https://www.ncbi.nlm.nih.gov/geo/query/acc.cgi?acc=GSE192499] and GSE192501 [https://www.ncbi.nlm.nih.gov/geo/query/acc.cgi?acc=GSE192501], respectively. Assemblies and assembly-based phased variant calls from Garg et al. were acquired from [ftp://ftp.dfci.harvard. edu/pub/hli/whdenovo/]. PacBio HiFi reads used in collapsed analyses were acquired from the GIAB Consortium: [ftp://ftp-trace.ncbi.nlm.nih.gov/ReferenceSamples/giab/data/NA12878/HudsonAlpha_PacBio_CCS/PBmixSequel851_2_B01_PCCL_30hours_15kbV2PD_70pM_HumanHG001_CCS/m64109_200815_033514.fastq.gz], [ftp://ftp-trace.ncbi.nlm.nih.gov/ReferenceSamples/giab/data/NA12878/HudsonAlpha_PacBio_CCS/PBmixSequel851_1_A01_PCCL_30hours_15kbV2PD_70pM_HumanHG001_CCS/m64109_200813_162416.fastq.gz], [ftp://ftp-trace.ncbi.nlm.nih.gov/ReferenceSamples/giab/data/NA12878/HudsonAlpha_PacBio_CCS/PBmixSequel846_3_C01_PCDB_30hours_15kbV2PD_70pM_HumanHG001_CCS/m64109_200808_191025.fastq.gz], [ftp://ftp-trace.ncbi.nlm.nih.gov/ReferenceSamples/giab/data/NA12878/HudsonAlpha_PacBio_CCS/PBmixSequel846_1_A01_PCCL_30hours_15kbV2PD_70pM_HumanHG001_CCS/m64109_200805_204709.fastq.gz]; [ftp://ftp-trace.ncbi.nlm.nih.gov/ReferenceSamples/giab/data/NA12878/HudsonAlpha_PacBio_CCS/PBmixSequel846_4_D01_PBXM_30hours_21kbV2PD_70pM_HumanHG001_CCS/m64109_200810_062248.fastq.gz] and [ftp://ftp-trace.ncbi.nlm.nih.gov/ReferenceSamples/giab/data/NA12878/HudsonAlpha_PacBio_CCS/PBmixSequel846_2_B01_PCCM_30hours_21kbV2PD_70pM_HumanHG001_CCS/m64109_200807_075817.fastq.gz]. IMGT/HLA database (v3.44) : [https://www.ebi.ac.uk/ipd/imgt/hla/]. The source data underlying Figure. 1b, 4e, 4g and Supplementary Figure.7c, 8c, 8d are provided as a Source Data file. Source data are provided with this paper.

Human research participants

Policy information about studies involving human research participants and Sex and Gender in Research.

Reporting on sex and gender	N/A
Population characteristics	N/A
Recruitment	N/A
Ethics oversight	N/A

Note that full information on the approval of the study protocol must also be provided in the manuscript.

Field-specific reporting

Please select the one below that is the best fit for your research. If you are not sure, read the appropriate sections before making your selection.

☒ Life sciences ☐ Behavioural & social sciences ☐ Ecological, evolutionary & environmental sciences

For a reference copy of the document with all sections, see nature.com/documents/nr-reporting-summary-flat.pdf

Life sciences study design

All studies must disclose on these points even when the disclosure is negative.

Sample size	No sample size calculation was required for our study, as only GM12878 cell line and 293T cell line were used.

Data exclusions	No data were excluded from our study.
Replication	All attempts at replication were successful. At least three biological replicates were performed independently for all experiments.
Randomization	For all cell culture and validation experiments, all samples were treated by the same researcher in parallel in random order to eliminated covariates.
Blinding	Experiments designed for validation was blinded during the Experiments. The experiments were blinded during the analyses for allele-specific expression and methylation.

Reporting for specific materials, systems and methods

We require information from authors about some types of materials, experimental systems and methods used in many studies. Here, indicate whether each material, system or method listed is relevant to your study. If you are not sure if a list item applies to your research, read the appropriate section before selecting a response.

Materials & experimental systems

n/a	Involved in the study
☒	☐ Antibodies
☐	☒ Eukaryotic cell lines
☒	☐ Palaeontology and archaeology
☒	☐ Animals and other organisms
☒	☐ Clinical data
☒	☐ Dual use research of concern

Methods

n/a	Involved in the study
☒	☐ ChIP-seq
☒	☐ Flow cytometry
☒	☐ MRI-based neuroimaging

Eukaryotic cell lines

Policy information about cell lines and Sex and Gender in Research

Cell line source(s)	GM12878 cell line was acquired from Coriell Institute for Medical Research. HEK 293T cell line was received from American Type Culture Collection (ATCC) repository.
Authentication	No additional authentication was performed on cell lines.
Mycoplasma contamination	All cell lines tested negative for mycoplasmas contamination.
Commonly misidentified lines (See ICLAC register)	No commonly misidentified cell lines were used in this study.
